# Demystifying Open Science in health psychology and behavioral medicine: a practical guide to Registered Reports and Data Notes

**DOI:** 10.1080/21642850.2024.2351939

**Published:** 2024-05-29

**Authors:** Emma Norris, Aoife O’Mahony, Rory Coyne, Tugce Varol, James A. Green, James Reynolds, Elaine Toomey

**Affiliations:** a Department of Health Sciences, Brunel University London, London, UK; b School of Psychology, Cardiff University, Cardiff, UK; c School of Psychology, University of Galway, Galway, Ireland; d Public Engagement and Science Communication Group, Freudenthal Institute, Utrecht University, Utrecht, The Netherlands; e Physical Activity for Health Research Centre, Health Research Institute (HRI) and School of Allied Health, University of Limerick, Limerick, Ireland; f School of Psychology, Aston University, Birmingham, UK; g Centre for Health Research Methodology, School of Nursing and Midwifery, University of Galway, Galway, Ireland

**Keywords:** Open Science, Open Scholarship, Open Research, Registered Reports, Data Notes, Health Psychology, Behavioral Medicine

## Abstract

Open Science practices are integral to increasing transparency, reproducibility, and accessibility of research in health psychology and behavioral medicine. Drives to facilitate Open Science practices are becoming increasingly evident in journal editorial policies, including the establishment of new paper formats such as Registered Reports and Data Notes. This paper provides: (i) an overview of the current state of Open Science policies within health psychology and behavioral medicine, (ii) a call for submissions to an Article Collection of Registered Reports and Data Notes as new paper formats within the journal of *Health Psychology & Behavioral Medicine*, (iii) an overview of Registered Reports and Data Notes, and (iv) practical considerations for authors and reviewers of Registered Reports and Data Notes.

Open Science, sometimes also referred to as open scholarship or open research, can be defined as a broad set of practices promoting transparency, reproducibility, replicability, and accessibility (Munafò et al., 2017; Parsons et al., 2022; Thibault et al., 2023). For researchers in particular, Open Science practices play a vital role throughout the entire research project lifecycle, spanning design, conduct, analysis, reporting, and publishing. In the design phase, preregistration enables researchers to meticulously plan their studies by registering hypotheses, study designs, and analysis plans either before the commencement of data collection or analysis (Bosnjak et al., 2022; Sarafoglou et al., 2022; Van den Akker et al., 2023; Van't Veer & Giner-Sorolla, 2016). During conduct, analysis, and reporting phases, researchers can adhere to reporting checklists (e.g. PRISMA for systematic reviews; Page et al., 2021 and STROBE for observational studies; Von Elm et al., 2007), publicly share data, materials, and code on platforms like the Open Science Framework (OSF; Foster & Deardorff, 2017), and follow FAIR (Findable, Accessible, Interoperable, and Reproducible) principles for data, code, and software (Wilkinson et al., 2016). In the publishing phase, open peer review, open access, and preprints can help promote transparency and accessibility of completed research (Fraser et al., 2021; Kathawalla et al., 2021; McKiernan et al., 2016). This proactive approach can contribute to efforts to reduce the likelihood of the number of intentional or unintentional questionable research practices, such as *p*-hacking or HARKing (Hypothesizing After the Results are Known) (Nosek et al., 2018). Preprints and Registered Reports (discussed in more detail later in this paper) also serve to reduce publication bias, which can lead to overestimates of effect sizes and decreases the value of meta-analyses where studies with null findings are less likely to be included (Dwan et al., 2013). Engaging with Open Science practices can therefore address key issues at all stages of the research process, thus improving rigor, transparency and potential for impact.

## Current progress towards Open Science in the fields of health psychology and behavioral medicine

Open Science practices are integral to health psychology and behavioral medicine to facilitate the evaluation, replication, and eventual adoption of effective interventions, designed for public and patient benefit (Norris & O’Connor, 2019; Zečević et al., 2020). Current levels of engagement with Open Science practices within the health psychology community are relatively unclear. A recent survey of Open Science practices within UK psychology researchers identified an overall mean engagement score in health psychology researchers of 4.01/7 (1 = Never, 7 = Always; *n* = 69; Silverstein et al., 2024): however, this is an extremely small sample. Clear progress is underway to promote Open Science practices within health psychology and behavioral medicine. In 2018, a Synergy Expert Meeting of 16 experts organized by the European Health Psychology Society (EHPS) identified three priority actions for opening up research in health psychology: (i) supporting researchers to make data open and understanding researchers’ beliefs and attitudes regarding open data; (ii) integrating Open Science in teaching curricula; (iii) expanding Open Science and open data policies within health psychology journals (Kwasnicka et al., 2021). To support these priority actions, the EHPS Open Science Special Interest Group was established in 2020 (Norris & Toomey, 2020) and is developing a suite of training and support resources for health psychology and behavioral medicine researchers in Open Science. The group also conducts primary research on Open Science within the field of health psychology, including a recent Delphi research prioritization exercise to establish consensus amongst EHPS members on a clear starting point for this research (Norris et al., 2022). The research priorities identified were: (i) To what extent are Open Science behaviors currently practiced in health psychology? (ii) How can we maximize the usefulness of Open Data and Open Code resources? (iii) How can Open Data be increased within health psychology? (Norris et al., 2022).

A recent statement from the Behavioral Medicine Research Council (BMRC: established in 2017 after unanimous approval from the Academy of Behavioral Medicine Research, the Society for Health Psychology, the American Psychosomatic Society and the Society of Behavioral Medicine) affirmed the importance of Open Science practices within health psychology and behavioral medicine to benefit health research and ensure societal impact (Segerstrom et al., 2023). Notably, this statement was published simultaneously across three leading journals in the field: *Annals of Behavioral Medicine* and *Psychosomatic Medicine* and *Health Psychology*, emphasizing the importance of these practices. The authors also conducted a brief analysis of Open Science behaviors within these three journals from 2018 to 2020, specifically the use of preregistration, Registered Reports, gold open access, protocol availability and statements on data and material availability. However, consistent with previous similar research (McVay et al., 2021), they found that overall practice of Open Science behaviors were low, except for the use of gold open access in one journal. Similarly, McVay and Conroy (2021) examined research presented at the 2019 Society of Behavioral Medicine annual meeting and found that approximately 26% of 1636 presentations reported using any Open Science practices (e.g. preregistration of study design or analysis, and materials or data sharing), and only 10% reported using more than one of these four practices. However, as highlighted by Norris et al. (2022), there has been limited meta*-*research to date that explores the practice of Open Science behaviors in the published literature within the related fields of health psychology and behavioral medicine.

Academic journals play a significant role in facilitating Open Science behaviors amongst researchers. For example, an interrupted time series by Hopewell et al. (2012) showed that journals with an active editorial policy regarding the use of CONSORT abstract reporting guidelines led to improved reporting of abstracts of randomized trials. Similarly, the Nature Publication Quality Improvement Project (NPQIP) Collaborative group (2019) found that changes in Nature journals’ editorial policies led to improvements in risk of bias reporting. In 2015, the Transparency and Openness Promotion (TOP) guidelines (Nosek et al., 2015) were developed to improve journal policies in this respect. TOP guidelines include eight modular standards for journal policies, including data citation, data, materials and code transparency, design and analysis, preregistration and replication, across three levels of engagement: disclose, require, or verify. However, despite the availability of these guidelines and the potential impact of journal policies on Open Science behaviors, several studies have shown that journals’ uptake of TOP guidelines and implementation of Open Science editorial policies in health and medical literature is low (Cashin et al., 2020; Gardener et al., 2022; Lee et al., 2018).

In recent times, several health psychology and behavioral medicine journals have generally shown progress towards becoming more open: adopting the Transparency and Openness Promotion (TOP) guidelines (Nosek et al., 2015) to varying degrees. Currently, some key health psychology journals require study pre-registrations for systematic reviews and meta-analyses (e.g. *Health Psychology Review*), randomized controlled trials (e.g. *Health Psychology*), and experimental studies (e.g. Psychology & Health). As of January 2024, *Health Psychology & Behavioral Medicine* requires preregistration for all interventions including feasibility studies. Some health psychology journals also currently offer Registered Report paper formats (e.g. *British Journal of Health Psychology, Psychology & Health*), while others have open peer review (e.g. *Health Psychology Review*). However, *Health Psychology Bulletin*, designed to be a disruptive fully Open Access journal, welcoming study replications and null finding reports as well as empirical papers and reviews (Peters et al., 2017), closed in June 2023 due to low submissions. Requirements in health psychology and behavioral medicine to submit data, code, and materials remain lacking, as are paper formats to document and describe the full range of research processes. Given the necessity of these different approaches in order to apply Open Science more widely, and to improve research transparency, reproducibility, replicability, and accessibility within health psychology and behavioral medicine, it is imperative for health psychology and behavioral medicine journals to ensure their policies facilitate these approaches.

## Call for article collection launching Registered Report and Data Note paper formats at *Health Psychology & Behavioral Medicine*

*Health Psychology & Behavioral Medicine* is pleased to now offer Registered Reports and Data Notes as new paper formats. The journal is also launching a call for submissions to an Article Collection celebrating the journal's launch of Registered Reports and Data Notes. Submissions of Stage 1 Registered Reports and Data Notes, adhering to the journal's author guidelines, are welcome for consideration. Full details on the call for this Article Collection are available here https://think.taylorandfrancis.com/article_collections/health-psychology-and-behavioral-medicine-registered-reports-and-data-notes-within-health-psychology-and-behavioral-medicine/.

This study received an ethical approval exemption from Brunel University London’s Ethics committee (47140-NER-Jan/2024- 49421-1).

## An introduction to Registered Reports and Data Notes for authors and reviewers

Here we provide specific methodological guidance regarding these two paper formats, which aim to increase accessibility and transparency of research in the field. This is not intended to be specific guidance about the current journal's guidelines for submission, as these guidelines may develop over time as methodologies improve and journal policies change. Instead we aim to provide an introduction about the current state-of-the-art for these submission types.

### Registered Reports

**Figure 1.**
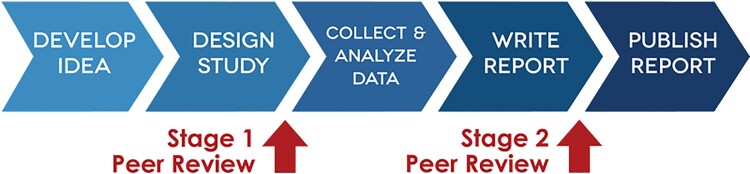
Registered Reports process. Figure reproduced from the Center for Open Science (www.cos.io/rr), without adaptations, under a CC-BY 4.0 license.

Registered Reports (RRs) are a form of empirical publication where study proposals are peer reviewed and may receive in-principle acceptance before data collection begins (Chambers & Tzavella, 2022). RRs hence expand the aforementioned benefits of preregistration, by additionally allowing preregistered research hypotheses, study designs, and analysis plans to be enhanced by peer review (Henderson & Chambers, 2022). Decisions on publication are therefore based on the research questions, theoretical basis and methods proposed, rather than the statistical significance or direction of effects identified in the study's results. The final (Stage 2) manuscript is reviewed again when complete, in order to assess the authors’ implementation of their pre-planned approach. See Figure 1 for an overview of this process. Open data and materials can, and arguably should, be published within the accepted Stage 2 Registered Report (Pennington, 2023).

The RRs format was first launched in several journals from approximately 2013 onwards, including *Cortex*, *Perspectives on Psychological Science*, and *Social Psychology* (Chambers & Tzavella, 2022). Ten years later, the format has now been offered at over 300 journals. While the majority of journals that offer RRs are focused on psychology, they have also been used in areas as diverse as business and economics and cancer biology (Center for Open Science, 2024; Montoya et al., 2021). The uptake of the format within health-related journals has been relatively limited to date, particularly within health psychology (Center for Open Science, 2024), although these are offered by the *British Journal of Health Psychology* and *Psychology & Health* and had also been offered by the now defunct *Health Psychology Bulletin*.

#### Advantages of Registered Reports

By conducting peer review before the data is collected, RR publication decisions are based on methodological quality, rather than the significance or novelty of findings (Chambers, 2013; Chambers, 2015). RRs also help to reduce publication bias, facilitating the publication of important null results. Scheel et al. (2021) reported that 66% of RRs had null results with respect to their first hypothesis, compared with just 4% of standard research reports. Additionally, having some peer review at this earlier stage of publication allows researchers to receive feedback when they really need it: at the design stage of their study before conducting it (Henderson & Chambers, 2022). A recent evaluation of RRs involved researchers reading the full-texts of psychology and neuroscience RRs and standard reports while blinded to whether it was a RR or not (Soderberg et al., 2021). The researchers then rated the papers for methodological quality across 19 criteria, with RRs being indistinguishable from or outperforming standard reports across all criteria.

Initiatives to maximize the value of RRs are also evident. Registered Report – Funding Partnerships have been trialed, whereby authors’ application for research project funding and for acceptance at a participating journal are considered simultaneously, reducing the work required to submit separate applications for each of these (Clark et al., 2021; Drax et al., 2021). Additionally, Peer Community In Registered Reports (PCI-RR; Peer Community-In, 2023a) is a non-profit platform launched to handle the reviewing of RRs and recommends these to participating journals for publication. This allows authors to choose which of the ‘PCI-friendly’ journals who opt-into the scheme they wish to publish their work in, and reduces the effort that would be required to submit to multiple journals individually (Pennington & Heim, 2022). ‘PCI-interested’ journals opt-in to be notified of Stage 1 Registered Reports accepted via PCI-RR for consideration of publication. There is currently a lack of PCI-RR friendly participation in journals related to health psychology and behavioral medicine. *Addiction Research and Theory* are currently a PCI-friendly journal (Pennington & Heim, 2022), however, this represents a very small area of interest within the wider context of health psychology and behavioral medicine.

#### Challenges of Registered Reports and potential solutions

Time required for Registered Report peer review is a common perceived barrier, particularly for projects with short timescales and researchers on short-term contracts and students (Allen & Mehler, 2019; Baldwin, 2024; Morey & Tzavella, 2018). Efforts have been made at some outlets to minimize waiting times, such as scheduled review track available through PCI-RR. Despite the challenges involved in conducting studies within limited timeframes, RRs have been demonstrated to be feasible for early career researchers, both in terms of the experience of publishing RRs during a PhD (Henderson, 2019), and also in the high proportion of RR submissions (77–78%) that are led by ECRs (Chambers & Tzavella, 2022). As RRs were originally designed for research which is primarily confirmatory (Chambers & Tzavella, 2022), extra support is arguably required for diverse paper formats. The following articles provide guidance and examples for exploratory (Chambers & Tzavella, 2022; McIntosh, 2017), qualitative research (Hunter et al., 2021; Karhulahti et al., 2023) and systematic reviews and meta-analyses (Kathawalla & Syed, 2021). From a peer reviewer's perspective, it may be seen that RRs also require additional time for study appraisal (Chambers & Tzavella, 2022). Stage 1 peer review could be seen as lengthy than typical peer review due to the important influence their comments can have on methods and analysis for the given project, with further confirmatory Stage 2 review also required in the future. However, arguably the extra impact that Stage 1 peer review feedback can have on improving a study can also be seen as a motivating factor to review RRs in the first place. A wider range of misconceptions and realities of Registered Reports can be found in Chambers and Tzavella (2022: Table 1).

**Table 1.** Practical considerations of Registered Reports.

For Authors	For Reviewers
Check your chosen journal and research institution's respective policies on when to apply for ethical approval (e.g. before or after Stage 1 acceptance) (Henderson & Chambers, 2022) Communicate any deviations from your accepted Stage 1 manuscript with the journal editor (Henderson & Chambers, 2022) Transparently document any changes by updating your preregistration or online repository documentation on Open Science Framework, or your chosen repository (Henderson & Chambers, 2022) Your introduction and methods section in your Stage 2 manuscript should not change unnecessarily from your approved Stage 1 manuscript (Henderson & Chambers, 2022)	At Stage 1, assess the scientific validity and rationale of the proposed research plan, as well as the soundness, feasibility and clarity of the proposed methodology and analysis (Peer Community-In, 2023b) At Stage 2, consider whether the data truly tests the originally proposed hypotheses, and whether the introduction, rationale, and stated hypotheses are the same as the Stage 1 submission (Peer Community-In, 2023b) At Stage 2, consider whether the authors adhered to the procedures outlined in the Stage 1 submission, and assess the appropriateness of justifications for any deviations (Peer Community-In, 2023b) Do not reject papers at Stage 2 submission based on the direction or significance of the results obtained (Chambers & Tzavella, 2022)

#### Practical considerations of Registered Reports for authors

**‘**Ten simple rules for writing a Registered Report’ (Henderson & Chambers, 2022) provides a useful overview of considerations when submitting an RR. Useful tips from this paper for authors new to RRs include checking your chosen journal and research institution's respective policies on when to apply for ethical approval (e.g. before or after Stage 1 acceptance). You should also communicate any deviations from your accepted Stage 1 manuscript with the journal editor, ideally also transparently documenting any changes by updating your preregistration or online repository documentation on Open Science Framework or other chosen repository. When preparing your Stage 2 manuscript after your study's completion, your introduction and methods section should not change unnecessarily from your approved Stage 1 manuscript. A helpful planner for RR writing and avoiding Stage 1 rejection is also available (Henderson & Chambers, 2022). Table 1 outlines the key points to consider for authors wishing to submit an RR.

#### Practical considerations of Registered Reports for reviewers

RRs differ from standard reports in the stage of the research process at which they receive primary peer review, i.e. prior to data collection (Kiyonaga & Scimeca, 2019). Reviewer comments therefore have an even more important role in the research process, having the potential to inform and influence a study's aims, methodology, and analysis plans (Chambers & Tzavella, 2022). PCI-RR has provided helpful guidance for reviewers of RRs (Peer Community-In, 2023b), which is useful for reviewers regardless of whether RRs are received via PCI-RR or traditional journal submission. Reviewer considerations for Stage 1 submissions from this guidance include assessing the scientific validity and rationale of the proposed research plan, as well as the soundness, feasibility and clarity of the proposed methodology and analysis (Peer Community-In, 2023b). Reviewer considerations for Stage 2 submissions include whether the data truly tests the originally proposed hypotheses, and whether the introduction, rationale, and stated hypotheses are the same as the Stage 1 submission. Whether or not the authors adhered to the procedures outlined in the Stage 1 submission should also be examined, with justifications for deviations also assessed for their appropriateness (Peer Community-In, 2023b). Crucially, papers must not be rejected at Stage 2 submission based on the direction or significance of the results obtained (Chambers & Tzavella, 2022). The key points for reviewers to consider are outlined in Table 1.

### Data Notes

**Figure 2.**
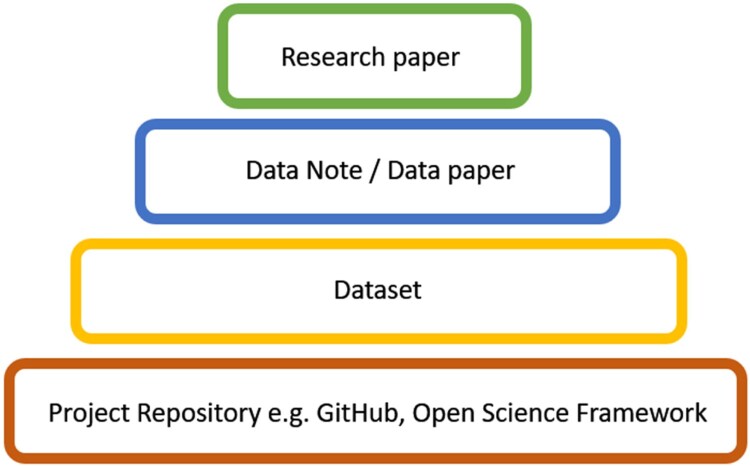
How Data Notes (also called Data Papers) fit within the research and dissemination ecosystem. Figure adapted from McGillivray et al. (2022).

Data Notes, sometimes also referred to as Data Papers (McGillivray et al., 2022), are an additional new class of articles offered by journals aiming to increase research and data transparency. Data Notes create the opportunity to describe a specific openly-available dataset in detail, with the purpose of making the data FAIR (Findable, Accessible, Interoperable and Reusable; Wilkinson et al., 2016). In writing a Data Note, authors produce clear citable documentation of data stored in an open repository, such as the Open Science Framework or GitHub (Figure 2). A Data Note typically includes an abstract, and an introduction to the dataset including the rationale for collecting the data, methods about how the data was collected, information about the quality of the data, limitations of the data, guidance on how to access the dataset, and links to any previous publications that make use of the data.

Data Notes do not include results, analysis or interpretations of the data. A Data Note should include the following details of the dataset:

- Where to find the dataset (i.e. the name of the repository) and its DOI (Digital Object Identifier)
- Who created or collected the data, and when
- What the format of the data is
- Explanations of variables within the dataset and their origins
- Any restrictions to the dataset's use.

Although currently predominantly found within life science research in venues such as *BMC Research Notes*, Data Notes are increasingly being used within public health. An example of a relevant study is Beyene's Data Notes paper on the effects of food insecurity on health outcomes (Beyene, 2023). It outlines the various open online data sources compiled from the Food and Agricultural Organization, the World Bank and the United Nations Development Programme to form the dataset, variables used within the analysis and details on how the data was analyzed within STATA in an Additional File.

However Data Notes are not yet prevalent within health psychology. The addition of Data Notes within *Health Psychology & Behavioral Medicine* provides a venue for researchers in the field to fully explain the processes behind their open datasets.

**Figure 3.**
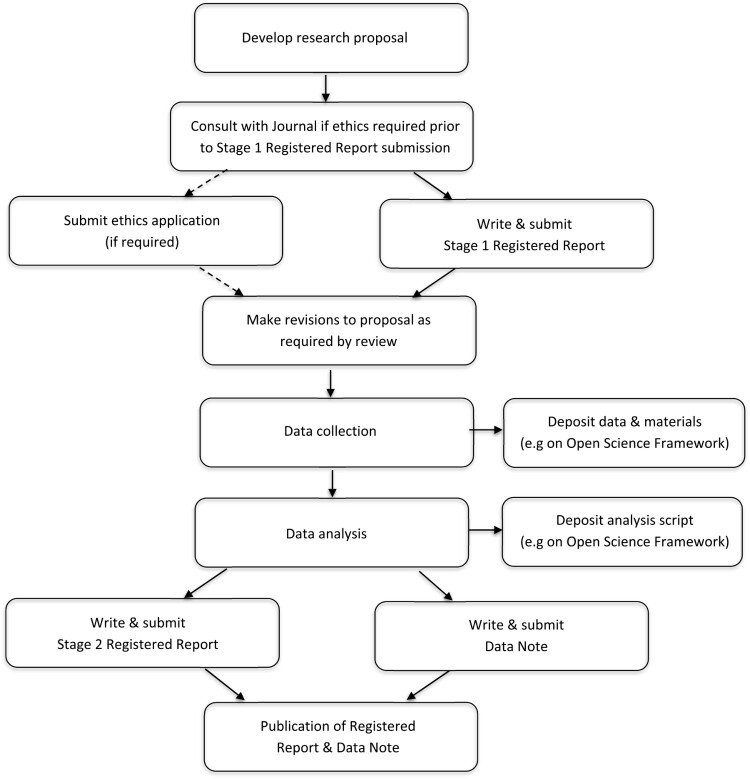
Research workflow for integrating Registered Reports and Data Notes within a study. Demonstration of how to integrate both Registered Reports and Data Notes within a study.

Figure 3 illustrates how both Registered Reports and Data Notes could be integrated into a single research study.

#### Advantages of Data Notes

Data Notes can be useful for multiple purposes. Within research, Data Notes are useful for adding additional detail to published data resources: providing depth limited to traditional paper formats. As a peer-reviewed publication, having external review of a dataset's documentation should improve the quality of the documentation. As an indexed and citable object, they increase the ability of other researchers to find your data set, as well as enhancing its potential for re-use. The additional visibility of your data can foster new collaborations, as well as making citation and attribution easy. Data Notes might be particularly useful for datasets that are available but have restricted access. By describing both the data and the access process, a data note should guide external users through the access process.

Within education, Data Notes can provide information on data collection and analysis decision making processes: useful for undergraduate and postgraduate studies, as well as for researchers wanting to develop their research skills.

#### Challenges of Data Notes and potential solutions

The primary challenge for Data Notes may be the fear of authors being ‘scooped’ by other researchers. This can be managed by delaying the submission of a Data Note until planned papers are preprinted or published. However, the risk of being scooped with your own data is perhaps overstated, and some researchers have found that the impact on having someone publish similar or the same findings has only a modest impact on citations (Callaway, 2019). Data uploaded online need not be automatically open to everyone. Open Science repositories have different access options available to specify who can access data and other documentation and when. Open Science Framework allows whole projects or components of projects to be made public or private so that only project collaborators can access them. Zenodo allows files to be deposited under closed, open or embargoed access.

A second challenge for Data Notes and Open Data more generally is the need for data access to be integrated into all ethical applications and participant informed consent documents (Branney et al., 2023). Hence, it is not permissible to make a dataset open or to subsequently publish a Data Note without participants having already consented to their data being made open. There are also considerations of de-identification and anonymization of data which will often need to be performed for types of data common in health psychology and behavioral medicine: such as data on rare disease populations (Branney et al., 2023; Norris et al., 2022).

#### Practical considerations of Data Notes for authors

The biggest practical question for authors is, if your data is already lodged in a repository, what is the additional return on your investment in publishing a Data Note? It perhaps makes the most sense for larger and more extensive datasets where there is more potential for further investigation, rather than as a routine practice. The effort in collecting, for example, a rich longitudinal dataset is often immense, so the additional effort of clear documentation through a Data Note, increasing its discoverability and reusability seems very clear. The Data Note must provide a description of the related dataset, including when, where, and how the data was collected (Taylor & Francis, 2024). Additional details that can be described include the names of all variables in the dataset, how each variable was measured, values for each number in the dataset, how new variables were generated within the dataset, and origins of measures used in the dataset (e.g. how existing measures that have been adapted and why). Further guidance on publishing Data Notes within Taylor & Francis journals is available here.

#### Practical considerations of Data Notes for reviewers

For peer reviewers, evaluating a Data Note changes some of the usual questions for a reviewer. Obviously, there are no results or conclusions to consider, but attention is then focused on how clearly and thoroughly the methods and dataset are described. Is the quality sufficient? Is the dataset substantive enough and of sufficient value to the research community to make re-use likely? Table 2 outlines the key points to consider for authors wishing to submit a Data Note.

**Table 2.** Practical considerations of Data Notes.

For Authors	For Reviewers
Consider the value of publishing a Data Note in addition to lodging your data in a repository. For example, clear documentation of a rich longitudinal dataset, for which the effort to collect the data has been immense, could be particularly beneficial (Taylor & Francis, 2024) Ensure that ethical approval and participant informed consent is granted on the basis that the resulting data can be made open. Ensure any requirements of de-identification and anonymization of data are performed, as required by ethical approval Aim to describe the data clearly and in such a way that it can be understood by a non-specialist reader (BMC, 2024). Description of the data includes when, where and how the data was collected Additional details that can be described include the names of all variables in the dataset, how each variable was measured, values for each number in the dataset, how new variables were generated within the dataset, and origins of measures used in the dataset When describing the data, do not include any interpretations of the data, analyzed results, or conclusions. The aim is to be purely descriptive (BMC, 2024)	Assess how clearly and thoroughly the methods and dataset are described Assess whether the data quality is sufficient, and whether the dataset is substantive enough and of sufficient value for the research community to make re-use likely

## Future directions for open science in health psychology and behavioral medicine

The embracing of Registered Reports and Data Notes by health psychology and behavioral medicine journals is a welcome advance in the move towards Open Science in the field. However, there is still much work to be done across research structures globally to further support and facilitate Open Science practices within health psychology and behavioral medicine. Journals have a crucial role to play and can further Open Science by implementing the TOP guidelines (Cashin et al., 2020; Nosek et al., 2015), including supporting and requiring authors to make their data and materials openly available alongside their paper. Journals representing the broad domains of health psychology and behavioral medicine are yet to join PCI-Registered Reports, either as PCI-friendly or PCI-interested. Having examples of journals publicly supporting the PCI scheme would indicate support for Registered Reports within this domain.

Beyond journals, institutions and their leaders also have a role to play in developing a supportive culture where Open Science is rewarded in funding, hiring and promotion criteria (Thibault et al., 2023). Meta-research into Open Science practices within health psychology and behavioral medicine is lacking (Norris et al., 2022): with large scope to apply behavior change to improve Open Science practices themselves (Norris & O’Connor, 2019). Established behavior change theories, such as the Behavior Change Wheel (Michie et al., 2011), are ready to aid the development and evaluation of Open Science interventions, as has already been provisionally done in the context of preregistration (Osborne & Norris, 2022). Open Science training must also be routinely embedded within graduate training programs: with outstanding Open Educational Resources now available to support this, such as PaPOR Trail (Egan et al., 2020; Pownall et al., 2021). Finally, international organizations representing research and practice in health psychology and behavioral medicine could do more to better support and advocate for Open Science, extending work of groups such as the European Health Psychology Society for Open Science Special Interest Group (Norris & Toomey, 2020).

## Data Availability

No data is associated with this paper.
